# Diversity of *Cryptosporidium* spp. in wild rodents from the Canary Islands, Spain

**DOI:** 10.1186/s13071-020-04330-9

**Published:** 2020-09-04

**Authors:** Katherine García-Livia, Aarón Martín-Alonso, Pilar Foronda

**Affiliations:** 1 Instituto Universitario de Enfermedades Tropicales y Salud Pública de Canarias, Universidad de La Laguna, San Cristobal de La Laguna, Canary Islands Spain; 2 Departament Obstetricia y Ginecología, Pediatría, Medicina Preventiva y Salud Pública, Toxicología, Medicina Legal y Forense y Parasitología, Universidad de La Laguna, San Cristobal de La Laguna, Canary Islands Spain

**Keywords:** *Cryptosporidium tyzzeri*, *Cryptosporidium meleagridis*, *Cryptosporidium muris*, Zoonoses, Wild rodents, Canary Islands

## Abstract

**Background:**

*Cryptosporidium* spp. are worldwide protozoan parasites which include species that can lead to cryptosporidiosis in humans. Different animal species can serve as reservoirs and sources of dissemination of the disease, such as rodent species due their potential in transmitting zoonotic pathogens to humans and other animals. In the Canary Islands (Spain), *Cryptosporidium parvum* and *Cryptosporidium hominis* have been identified in patients with diarrhea. However, the occurrence of *Cryptosporidium* spp. in possible reservoirs in this archipelago remains unclear. Considering the zoonotic potential of these protozoans, the aim of the present study was to determine the presence of *Cryptosporidium* spp. in peridomestic wild rodents and the possible role of these mammals as a source of transmission of these protozoans in Canary Islands.

**Methods:**

A total of 179 rodents belonging to *Rattus rattus* and *Mus musculus domesticus* from four Canary Islands, La Palma, El Hierro, Tenerife and Lanzarote, were analyzed. Feces were screened for *Cryptosporidium* spp. by nested PCR of the *18S* ribosomal RNA fragment and the sequences used for phylogenetic analyses.

**Results:**

*Cryptosporidium* spp. were found widely distributed with an overall prevalence of 12.30% in rodents (13.86% for *R. rattus* and 10.25% for *M. m. domesticus*). The overall prevalence by island was 19.60% for Tenerife, 7.14% for La Palma, 5.71% for El Hierro and 0% for Lanzarote. *Cryptosporidium tyzzeri*, *Cryptosporidium meleagridis*, *Cryptosporidium muris* and *Cryptosporidium* sp. rat genotype I and II/III were successfully identified, in addition to two unidentified *Cryptosporidium* genotypes.

**Conclusions:**

This study contributes to the knowledge of the biodiversity and distribution of *Cryptosporidium* spp. in wild rodents from the Canary Islands, highlighting the presence of three zoonotic species, *C. tyzzeri*, *C. meleagridis* and *C. muris*, being the first detection of these three species in wild rodents in the Canary Islands and the first report of *C. meleagridis* in *R. rattus*. Given the results obtained in our study, future studies in non-sampled areas are required to better understand the epidemiology of these protozoans in wild rodents in the archipelago.
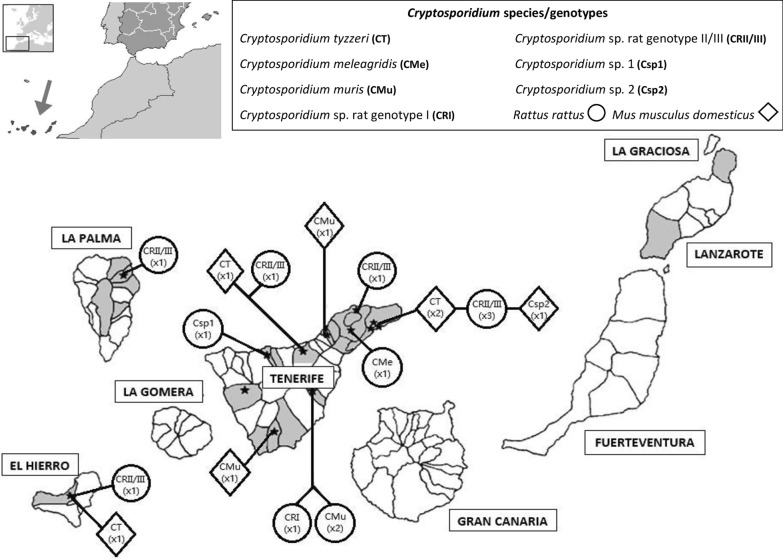

## Background

*Cryptosporidium* spp. are worldwide protozoan parasites that can be found in the environment and parasitizing humans and an extensive group of wild animal species [1]. They are considered common causes of water-borne and food-borne outbreaks and have been identified in different groups of domestic and wild animal species such as pets, livestock and rodents, among others [2–6]. *Cryptosporidium* species are resistant to various environmental conditions and are opportunistic pathogens responsible of cryptosporidiosis, a gastrointestinal disease with a wide spectrum of clinical symptoms, where the severity and persistence of the infection are dependent on host factors and parasite characteristics [7].

In humans, *Cryptosporidium* spp. can cause cryptosporidiosis, a disease with mild to severe signs and symptoms depending on parasite-related factors, site of infection and nutritional and immune status of the host [8]. In healthy individuals, cryptosporidiosis is a self-limiting diarrhea and may also result in chronic or life-threatening diarrhea in individuals with a compromised immune system [8]. *Cryptosporidium parvum* and *Cryptosporidium hominis* are the most frequent species related to cryptosporidiosis and the most common species involved in water-borne epidemics [9, 10]. *Cryptosporidium hominis* is responsible for approximately 80% of human cases in European countries [11], and the main species causing illness in childhood (see [8]).

More than 40 *Cryptosporidium* species have been described as valid worldwide [12–14], and around 20 species and genotypes have been reported in humans, such as *Cryptosporidium meleagridis*, *Cryptosporidium felis*, *Cryptosporidium canis*, *Cryptosporidium ubiquitum*, *Cryptosporidium cuniculus*, *Cryptosporidium viatorum*, *Cryptosporidium muris*, *Cryptosporidium andersoni*, *Cryptosporidium suis*, *Cryptosporidium bovis*, *Cryptosporidium xiaoi*, *Cryptosporidium erinacei*, *Cryptosporidium fayeri*, *Cryptosporidium scrofarum*, *Cryptosporidium tyzzeri*, chipmunk genotype I, horse genotype, skunk genotype and mink genotype [12, 15].

Wild mammals, especially rodents, can coexist in anthropogenic environments and pose a risk to public health as they are reservoirs of viruses, bacteria and parasites [16], many of them with zoonotic importance, including some *Cryptosporidium* species, among others [17]. There are hosts of a large number of *Cryptosporidium* species and genotypes that have been diagnosed in sporadic human cases including *C. parvum*, *C. muris*, *C. ubiquitum*, *C. meleagridis*, *C. scrofarum*, *Cryptosporidium proliferans*, *Cryptosporidium occultus*, *C. viatorum*, *C. canis*, *Cryptosporidium wrairi*, *C. tyzzeri*, *Cryptosporidium rubeyi*, *C. andersoni*, *C. hominis*, *C. suis* and rat genotypes I–IV, mouse genotypes II and III, the Naruko genotype, ferret genotype, chipmunk genotypes, hamster genotype, deer mouse genotypes I-IV, vole genotype, bear genotype, muskrat genotypes I and II and ground squirrel genotypes I–III [18–20]. Furthermore, recently some new species have been described in different rodent species, among them *Cryptosporidium alticolis* and *Cryptosporidium microti* in wild-caught common voles [21], *Cryptosporidium ditrichi* and *Cryptosporidium apodemi* in *Apodemus* spp. mice [22, 23]. Among these recently discovered species, *C. ditrichi* has shown a certain zoonotic potential since it has been newly reported infecting three patients in Sweden [24].

Rodents adapt easily to a wide variety of habitats in abundant populations being present in more than 80% of the islands worldwide [25]. The Canary Islands, an archipelago composed by eight islands and islets, contain a marked vegetation and the weather is strongly influenced by humid trade winds producing a marked vegetations belts [26]; this favors the existence of many types of ecosystems where rodents can proliferate and increase their population easily.

It has been shown that the bioclimatic conditions affect the distributions of some pathogens in the Canary Islands [27]. The available data about the presence of *Cryptosporidium* species in this archipelago are based on human feces [28], wastewater samples [29–31] and birds, concretely in pigeons [30, 32], *C. hominis* and *C. parvum* being the frequently detected species.

Studies related to *Cryptosporidium* spp. in wild mammals as possible reservoirs in the Canary Islands are scarce. In case of wild rodents, the study carried out by Feliu et al. [33] was the first to provide data about the parasite fauna of both murine species studied, *Rattus rattus* and *Mus musculus domesticus*, in El Hierro Island, and revealed an overall prevalence of *C. parvum* of 48.6%.

Therefore, the aim of the present study was to analyze the distribution, prevalence and identity of *Cryptosporidium* species present in wild rodents from the Canary Islands, and analyze the role of wild rodents in the maintenance and transmission of *Cryptosporidium* spp. in this archipelago, with special attention to zoonotic species.

## Methods

### Sample collection

**Fig. 1:**
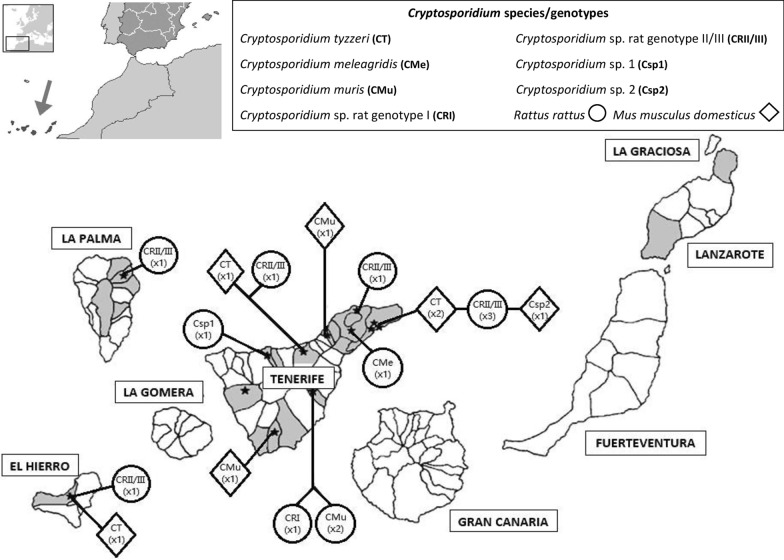
Location of the Canary Islands and the geographical distribution of rodents sampling locations analyzed in the present study (gray). Black stars represent the presence of *Cryptosporidium* spp. in wild rodents. An indication code for species, genotypes and hosts is provided at the top of the panel

The Canary Islands (Spain) are located off NW Africa, between 13° 23ʹ and 18° 8ʹ W and 27° 37ʹ and 29° 24ʹ N. From a multidisciplinary study carried out in this archipelago, a total of 179 wild rodents of the species *R. rattus* (*n* = 101) and *M. m. domesticus* (*n* = 78), from El Hierro (*n* = 35), La Palma (*n* = 14), Tenerife (*n* = 97) and Lanzarote (*n* = 33), were selected and analyzed for the presence of *Cryptosporidium* spp. (Fig. 1). A portion of fecal samples from the rectum of each animal has been preserved in 2.5% potassium dichromate and examined. Fecal oocysts were concentrated using a modification of Richie’s formaldehyde-ether method [34], in which the formaldehyde-ether was replaced by ethyl acetate, since it presents less distortion in the structures [35].

### DNA isolation

Total DNA of the concentrated feces samples was extracted following the manufacturer’s instructions with the commercial FastDNA^®^ Spin Kit for Soil (MP Biomedicals, Solon, OH, USA) using the homogenizer FastPrep-24™ 5G (MP Biomedicals) as oocyst disruptor. Quantity and quality of the extracted DNA were determined with the spectrophotometer Nanodrop ND-1000 (Thermo Fisher Scientific, Wilmington, DE, USA). DNA was stored at – 20 °C until further processing.

### PCR amplification

*Cryptosporidium* spp. were detected by nested PCR amplification of an 830 bp region of the *18S* ribosomal RNA fragment using the primers SSU-F1 and SSU-R1 for the primary PCR, and SSU-F2 and SSU-R2 for the secondary PCR [36]. The set-up of the PCR reactions was carried out following [37]. The reaction mixture for all pairs of primers contained 0.625 U *Taq* DNA polymerase, 0.4 *Taq* of each primer, 200 µM each dNTPs, 2 mM MgCl_2_, 1× buffer (Mg^2+^ free), 2 µl of DNA template and H_2_O to a total volume of 25 µl. The cycling conditions were initial denaturation of 95 °C for 5 min followed by 35 cycles 94 °C for 45 s, 45 s at suitable temperature (55 °C for the primary PCR and 58 °C for the secondary PCR), and 1 min at 72 °C, followed by a final extension step at 72 °C for 10 min. Both positive and negative controls were included in each test using *Cryptosporidium* sp. rat genotype III as a positive control. All amplicons were resolved on 1.5% agarose gels and purified using the EZNA Gel Extraction Kit (Omega Bio-Tek, Norcross, GA, USA).

### Sequencing and phylogenetic analyses

Purified products were sequenced in SEGAI (University of La Laguna sequencing services, La Laguna, Tenerife, Spain) and Macrogen Inc. (Madrid, Spain). Nucleotide sequences obtained for each amplified region were edited with the MEGA X program [38] and subsequently aligned with the ClustalW program included in MEGA X. Minor corrections, to increase the aligned sequence similarity and improve the inferences on any positional homology, were then made by hand. A BLAST search was carried out in order to elucidate any homologies or similarities of the nucleotide variants obtained with the sequences previously published in the GenBank database. The molecular identification was achieved by phylogenetic analysis through the Neighbor-Joining distance method [39] with at least 1000 bootstrap replications. For the phylogenetic analyses, the nucleotide sequences obtained in this work and other *Cryptosporidium* spp. sequences obtained from GenBank were aligned, *Toxoplasma gondii* was used as the outgroup.

Chi-square test was used to determine differences in the prevalence of *Cryptosporidium* spp. between the rodent species, and *P*-values below 0.05 were considered statistically significant.

## Results

The overall prevalence of *Cryptosporidium* spp. in rodents from the Canary Islands was 12.30%, since amplification of the *18S* rRNA gene fragment of *Cryptosporidium* spp. was successful in 22 out of 179 individuals tested. Most of the positive results were obtained on the island of Tenerife (19.60%) followed by La Palma (7.14%) and El Hierro (5.71%). All fecal samples tested for Lanzarote Island were negative (*n* = 33) (Table 1).

**Table 1:** Occurrence of *Cryptosporidium* species/genotypes in wild rodents in the Canary Islands, Spain

Island	*Rattus rattus* *n*/*N* (P%)	*Mus musculus domesticus* *n*/*N* (P%)	Total *n*/*N* (P%)	Species/Genotype
El Hierro	1/14 (7.14)	1/21 (4.76)	2/35 (5.71)	*Cryptosporidium tyzzeri* (M)
				*Cryptosporidium* sp. rat genotype II/III (R)
La Palma	1/9 (11.11)	0/5 (0)	1/14 (7.14)	*Cryptosporidium* sp. rat genotype II/III (R)
Tenerife	12/66 (18.18)	7/31 (22.60)	19/97 (19.60)	*Cryptosporidium muris* (R, M)
				*Cryptosporidium tyzzeri* (M)
				*Cryptosporidium. meleagridis* (R)
				*Cryptosporidium* sp. rat genotype I (R)
				*Cryptosporidium* sp. rat genotype II/III (R)
				*Cryptosporidium* sp. 1 (R)
				*Cryptosporidium* sp. 2 (M)
Lanzarote	0/12 (0)	0/21 (0)	0/33 (0)	
Total	14/101 (13.86)	8/78 (10.25)	22/179 (12.30)	

*Abbreviations*: *n*/*N* (P%), no. of positive animals for *Cryptosporidium* spp./no. of samples analyzed (prevalence %); R, *Rattus rattus*; M, *Mus musculus domesticus*

Considering the rodent species analyzed, both were found infected with *Cryptosporidium* spp., and no significant differences were found in relation to overall prevalence. The overall prevalence of infection with *Cryptosporidium* spp. in *R. rattus* was 13.86%, found in three of the four sampled islands (18.18% in Tenerife, 11.11% in La Palma and 7.14% in El Hierro). Considering *M. m. domesticus*, the overall prevalence was 10.25%. Mice parasitized by *Cryptosporidium* spp. were only found in Tenerife (22.60%) and El Hierro (4.76%) islands.

A total of 20 PCR-positive samples from La Palma, El Hierro and Tenerife islands were successfully sequenced. We decided to exclude one *Cryptosporidium* spp.-positive sample from the data used for genetic analyses due to an insufficient amount of DNA. The obtained sequences generated in this study have been submitted to the GenBank database under the accession numbers MN599007-MN599026 and MN783636-MN783642.

**Fig. 2:**
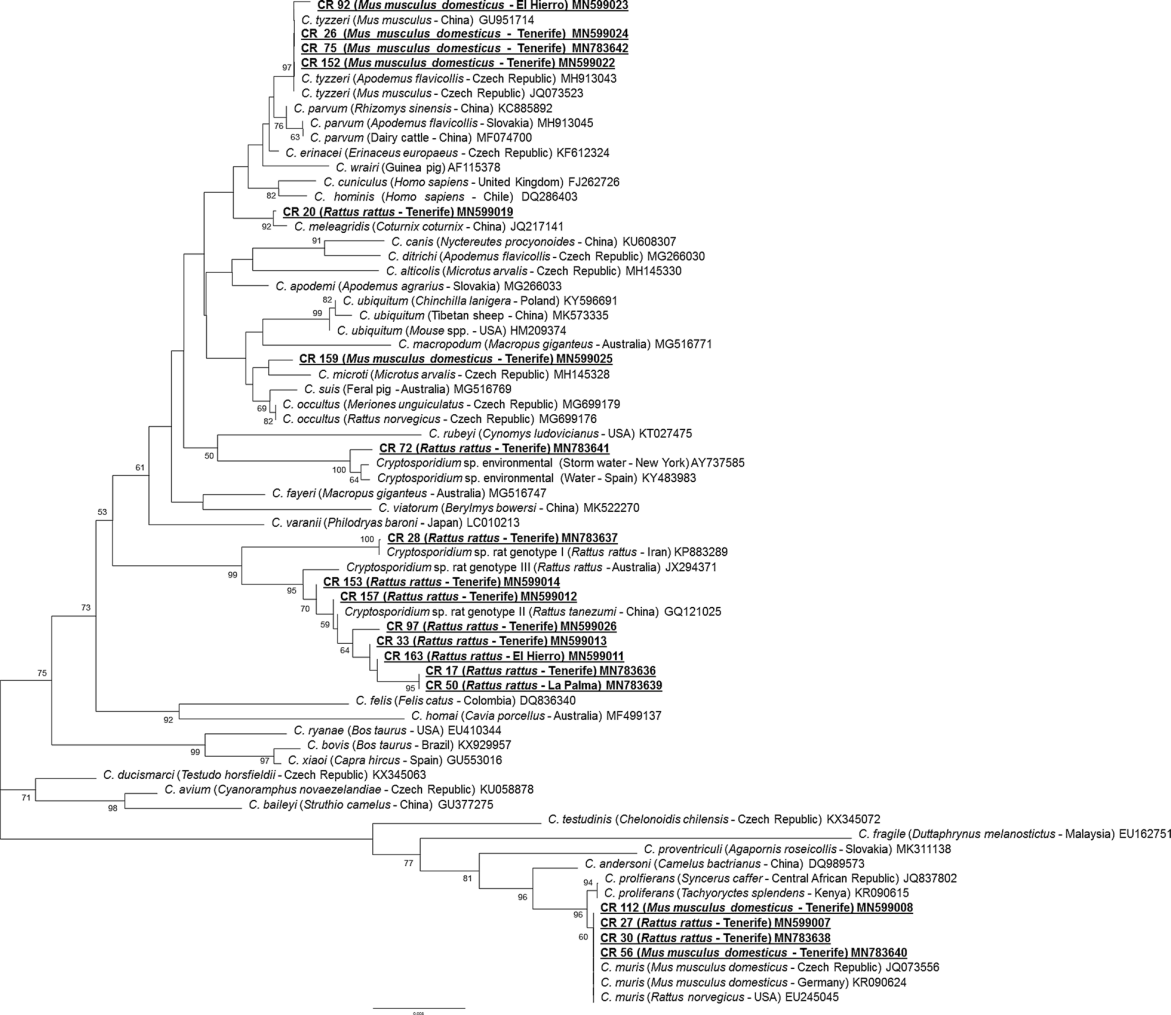
Phylogenetic analysis using the Neighbor-Joining method with p-distance and 1000 bootstrap replications based on the *18S* ribosomal RNA fragment (830 bp). *Toxoplasma gondii* was used as the outgroup (not included in the figure)

The Neighbor-Joining analysis of various *Cryptosporidium* species/genotypes and the 19 isolates from wild rodents from the Canary Islands are shown in Fig. 2. Phylogenetic analysis showed the presence of 7 *Cryptosporidium* species and genotypes (Fig. 2, Table 1). *Cryptosporidium tyzzeri* (*n* = 4), *C. meleagridis* (*n* = 1), *C. muris* (*n* = 4), *Cryptosporidium* sp. rat genotype I (*n* = 1) and *Cryptosporidium* sp. rat genotype II/III (*n* = 7) were identified. Furthermore, two *Cryptosporidium* genotypes with uncertain species status were found; the first, *Cryptosporidium* sp. 1 (isolate CR 72 from *R. rattus*), clustered with *Cryptosporidium* spp. sequences obtained from environmental samples in Spain (GenBank: KY483983) and in New York (GenBank: AY737585), and the other unnamed species, *Cryptosporidium* sp. 2 (isolate CR 159 from *M. m. domesticus*), grouped together with *C. microti* (GenBank: MH145328), and also clustered together with the species *C. suis* (GenBank: MG516769) and *C. occultus* (GenBank: MG699176 and MG699179).

*Cryptosporidium tyzzeri* was confirmed in 4 samples of *M. m. domesticus*, one from El Hierro island (isolate CR 92) and 3 from Tenerife island (isolates CR 26, CR 75 and CR 152). Another sequence obtained from *R. rattus* in Tenerife (isolate CR 20) clustered with *C. meleagridis*. Furthermore, phylogenetic analysis revealed that 8 sequences were broadly grouped in the clade containing rat genotypes I, II and III. One sequence (isolate CR 28 from Tenerife) clustered with rat genotype I in a separate clade, and the other 7 sequences (isolates CR 17, CR 33, CR 97, CR 153, CR 157 from Tenerife, CR 50 from La Palma and CR 163 from El Hierro) clustered with rat genotype II and rat genotype III. Moreover, 4 samples from Tenerife, 2 isolates from *R. rattus* (CR 27 and CR 30) and 2 isolates from *M. m. domesticus* (CR 56 and CR 112) were clustered with *C. muris* in the same clade.

## Discussion

Anthropogenic environmental changes, together with globalization effects can lead to an increase of zoonotic diseases whose origin is mostly wildlife [40, 41]. An example is cryptosporidiosis, considered one of the most common causes of diarrhea worldwide [42], in which wild rodents, due to their high reproductive capacity, zoonotic potential and close association with humans, favor the transmission by spreading through their feces the causative agents. This is considered a risk to both public and veterinary health as these animals could spread the disease to new environments and hosts [43, 44].

Prevalence rates of *Cryptosporidium* spp. infections in rodent species could be highly variable worldwide. In this sense, prevalence values are reported ranging between 8.0–31.4% and 2.1–63.0% in mice and rats, respectively [18, 19, 45, 46]. In our study, the overall prevalence of *Cryptosporidium* spp. in the Canary Islands was 10.25% in mice and 13.86% in rats. It is important to note that the prevalence values for *Cryptosporidium* spp. can fluctuate due to factors such as the host species analyzed, sample areas, bioclimatic conditions and methods applied [46].

In the Canary Islands, a total of 7 *Cryptosporidium* species/genotypes were detected in wild rodents in this study, demonstrating a high diversity of these protozoans in the archipelago. Previous studies carried out in this region have identified *C. parvum* and *C. hominis* in hospitalized patients with diarrhea [28], and *C. hominis* was also detected in other samples such as wastewater and pigeons feces [30].

In the present study, *C. tyzzeri*, *C. meleagridis* and *C. muris* were found in wild rodents, and considering their zoonotic potential, these species could be involved in cases of human diarrhea without specific identification, since the specific identification is not carried out at the Canary Hospitals.

*Cryptosporidium tyzzeri*, redescribed from *Cryptosporidium* sp. mouse genotype I [47], mostly infects domestic mice and small rodents, and it has been found in several non-specific hosts such as the lesser panda, black leopards, voles, snakes and horses, among others [48–51]. *Cryptosporidium tyzzeri* has also been recently reported for the first time in *Apodemus* spp., suggesting this murine species as a minor host due to the low prevalence obtained [23]. In humans, a severe cryptosporidiosis caused by a coinfection of *C. tyzzeri* and *C. parvum* has been reported in healthy young individual, demonstrating that the transmission of *Cryptosporidium* spp. from synanthropic rodents to humans can occur, suggesting the zoonotic potential of *C. tyzzeri* [52].

*Cryptosporidium meleagridis* is a common cause of cryptosporidiosis in avian hosts. Until now, studies carried out in Japan, New York and Malaysia, have reported the presence of *C. meleagridis* in *Rattus norvegicus* (brown rat), *Peromyscus* sp. (deer mouse), and in one unidentified rodent species [53–55]. Therefore, our study is the fourth report of *C. meleagridis* in rodents, and also the first report of *C. meleagridis* in *R. rattus*. As Tan et al. [55] suggest, this could indicate a possible role of rodents in mechanical transmission of this pathogen. *Cryptosporidium meleagridis* is considered to be the third most common species involved in human cryptosporidiosis after *C. hominis* and *C. parvum* [56]. It has shown that *C. meleagridis* is responsible for 10–20% of human cryptosporidiosis in some countries such as Thailand and Peru [57, 58]; zoonotic transmission in Sweden has also been reported [59]. In Spain, only one case of cryptosporidiosis caused by *C. meleagridis* has been reported [60].

In mainland Spain, previous studies in wildlife have demonstrated the presence of *C. muris* in rodents [61]. In different studies carried out worldwide, *C. muris* was reported in many rodent species [23, 46, 49, 62], and also in humans [61, 63–66], bilbies [67], birds [68] and other mammals [45, 49, 51, 69–72]. Moreover, *C. muris* has been reported in children and HIV-positive individuals from developing countries; however, healthy adults are also susceptible to infection [73].

*Cryptosporidium* sp. rat genotype I and II/III were also detected in the Canary Islands. As Zahedi et al. [74] have described in their review, these genotypes are host-adapted being cited only in rats. In the present study, this host specificity was also observed. Our phylogenetic analysis was not able to distinguish between genotypes II and III, probably due to genetic similarity between these genotypes at the *18S* locus, as previously published Ng-Hubling et al. [62].

Rat genotype I was identified in brown rats in China and Japan, in *Boa constrictor* in the USA and rats in the Philippines, and also in environmental samples such as wastewater in China and the UK, among others [18, 62, 75–77]. Rat genotype II has been previously reported in Asian house rats in China [78], wild black rats in northern Australia [79], Asian house rats and brown rats in the Philippines [62], and has also been found in sheep in Australia [80], among others. In the case of Rat genotype III, previous epidemiological studies highlight it as the most frequent genotype in rats [19]. In addition to rats, mice and cats have also been reported infected with *Cryptosporidium* sp. rat genotype III [62, 78, 79, 81].

The results obtained in our study suggest that *Cryptosporidium* sp. 1 may correspond to one unnamed *Cryptosporidium* genotype isolated from an environmental sample in Aragon (north-eastern Spain) and another unnamed genotype isolated from a storm event water sample from New York [82, 83]. It was not possible to identify to the species level *Cryptosporidium* sp. 2. However, the results obtained in our study seem to indicate that this isolate is more related to *C. microti*, *C. suis* and *C. occultus*; therefore, more studies are required to confirm the identity of this isolate. This finding may indicate the presence in the Canary Islands of a new species not previously described; however, more studies are required to confirm this hypothesis.

The results obtained in this study reflect a wide distribution of *Cryptosporidium* spp. in the Canary Islands. This work is of great interest since it constitutes the first finding in this archipelago of three zoonotic species, *C. tyzzeri*, *C. meleagridis* and *C. muris*. Most of the positive samples were found in Tenerife, being spread throughout the island including rural and metropolitan areas. The presence of zoonotic species could imply a risk of zoonotic transmission to humans.

## Conclusions

This study contributes to the knowledge of the biodiversity and distribution of *Cryptosporidium* species in wild rodents from the Canary Islands. Seven *Cryptosporidium* species/genotypes were identified, including a possible new species, demonstrating a high diversity of these protozoans in these islands. Also, host specificity previously described was observed for rat genotypes. This is the first detection of the zoonotic species *C. tyzzeri*, *C. muris* and *C. meleagridis* in the Canary Islands, *C. meleagridis* being the first report in *R. rattus*, as well as of *Cryptosporidium* sp. rat genotype I and II/III in this archipelago. *Cryptosporidium* spp. were found with wide distribution in wild rodents in the Canary Islands, including rural and metropolitan areas. Given the interesting results obtained in this study, further analysis in unsampled islands is required in order to better understand the epidemiology of *Cryptosporidium* spp. across the Canary Archipelago.

## Data Availability

Data supporting the conclusions of this article are included within the article. The newly generated sequences were deposited in the GenBank database under the accession numbers MN599007–MN599026 and MN783636–MN783642. The raw datasets analyzed by the authors during the present study are available from the corresponding author upon reasonable request.

## Abbreviations

SEGAI: Servicios Generales de Apoyo a la Investigación de la Universidad de La laguna
MEGA: Molecular Evolutionary Genetics Analysis
BLAST: Basic Local Alignment Search Tool
